# How to Improve Surveillance Program for Shiga Toxin-Producing *E. coli* (STEC): Gap Analysis and Pilot Study

**DOI:** 10.3390/pathogens13060511

**Published:** 2024-06-17

**Authors:** Valerio Massimo Sora, Francesca Zaghen, Alfonso Zecconi

**Affiliations:** 1 One Health Unit, Department of Biomedical, Surgical and Dental Sciences, School of Medicine, University of Milan, Via Pascal 36, 20133 Milan, Italy; 2 Department of Clinical and Community Sciences, School of Medicine, University of Milan, Via Celoria 22, 20133 Milan, Italy

**Keywords:** *E. coli*, STEC, epidemiology, surveillance, molecular biology, PCR, dairy, bovines

## Abstract

Several pathotypes of enteric *E. coli* have been identified. The group represented by Shiga toxin-producing *E. coli* (STEC) is of particular interest. Raw milk and raw milk products are significant sources of STEC infection in humans; therefore, identifying pathogens at the herd level is crucial for public health. Most national surveillance programs focus solely on raw milk and raw milk cheeses that are ready for retail sale, neglecting the possibility of evaluating the source of contamination directly at the beginning of the dairy chain. To assess the viability of the application of new molecular methodologies to STEC identification in raw milk filters and in calf feces, we analyzed 290 samples from 18 different dairy herds, including 88 bulk tank milk (BTM), 104 raw milk filters (RMF), and 98 calf feces samples. In total 3.4% of BTM, 41.4% of RMF, and 73.4% of calves’ feces were positive for *stx*, supporting our hypothesis that BTM is not a suitable matrix to assess the presence of STEC at herd level, underestimating it. Our conclusion is that the surveillance program needs critical and extensive improvements such as RMF and calves’ feces analysis implementation to be more efficient in detecting and preventing STEC infections. The epidemiology of these infections and the characteristics of the pathogen clearly show how a One Health approach will be pivotal in improving our capabilities to control the spread of these infections.

## 1. Introduction

### 1.1. Escherichia coli

*E. coli* is a gram-negative bacterium, facultative anaerobe, not sporogenous, belonging to the family *Enterobacteriaceae* [1]. The majority of animal species’ intestinal flora contain *E. coli* as the primary facultative anaerobe, which is often free of pathogenicity. However, many strains have evolved pathogenetic processes that enable them to cause a variety of illnesses in both humans and animals, including some extremely serious ones [2].

*E. coli* can be classified into pathotypes based on their pathogenetic profile, which considers the virulence factors, the diseases caused, and the phylogenetic profile [3]. Among *E. coli* causing enteric diseases, several pathotypes have been identified, namely intestinal pathogenic *E. coli* (IPEC), which includes enteropathogenic *E. coli* (EPEC), enterohemorrhagic *E. coli* (EHEC), enterotoxigenic *E. coli* (ETEC), enteroaggregative *E. coli* (EAEC), diffusely adherent *E. coli* (DAEC), enteroinvasive *E. coli* (EIEC), and extraintestinal pathogenic *E. coli* (ExPEC), which includes uropathogenic *E. coli* (UPEC), neonatal meningitis *E. coli* (NMEC), sepsis-associated *E. coli* (SEPEC), avian pathogenic *E. coli* (APEC), and mammary pathogenic *E. coli* (MPEC) [4].

Among the different pathotypes, the group represented by Shiga toxin-producing *E. coli* (STEC) is of particular interest. This group includes strains that produce at least one member of a class of potent cytotoxins called Shiga toxins. STEC, also called Verotoxin producing *E. coli* (VTEC), are named after the Shiga toxin (Stx), which is very similar to a cytotoxin produced by *Shigella dysenteriae* serotype 1 [5].

Among STEC strains, those having particular pathogenicity for humans are often also referred to as enterohemorragic *E. coli* (EHEC). This pathotype is a zoonotic agent that causes a potentially fatal human illness whose clinical spectrum includes bloody diarrhea, hemorrhagic colitis (HC), and hemolytic uremic syndrome (HUS) [6]. Since 1982, among STEC strains, EHEC has been a major source of food safety concern. The first strain included in this group is *E. coli* serotype O157: H7, which is still the most widespread EHEC serotype in the United States of America and Europe [7].

*E. coli* serotype O157:H7 is mostly associated with outbreaks and sporadic cases of HC and HUS in many countries; however, non-O157 STEC have been implicated in outbreaks around the world, and the number of reported cases has steadily increased every year. The Center for Disease Control and Prevention (CDC) has identified six other O groups, besides O157, to be of growing concern for public health and that are responsible for 71% of all illnesses caused by STEC: O26, O45, O103, O111, O121, and O145 (“Big 7”) [8]. The European Food and Safety Authority (EFSA) has identified five serogroups, O26, O103, O111, O145, O157 (“Big five”) [7], as being of major concern to human health in Europe. Currently, considerable attention is drawn to non-O157 STEC strains, particularly after the occurrence of a severe foodborne outbreak in 2011 in Germany caused by consumption of sprouts contaminated by STEC O104:H4 [9,10,11,12,13,14]. Nevertheless, following what has been stated in the 2020 EFSA risk assessment, we must not consider the serogroup and the presence of the *eae* gene as predictors of pathogenicity and clinical outcomes [15].

### 1.2. Reservoirs

Ruminants, especially cattle, are a major reservoir of a diverse group of STEC despite not being a source of diseases for these animals. Indeed, cattle are asymptomatic excretors of STEC, which are permanent or transient members of their normal intestinal flora [16].

Only the gastrointestinal tract of ruminants can be considered as a reservoir for these bacteria. The outbreaks investigated from 1982 to now have highlighted how ruminants, and the bovine species in particular, are almost always involved in the transmission of these bacteria to humans [16].

The persistence of STEC in individual animals is due to the ability of these bacteria to colonize specific portions of the gastrointestinal tract. The different interactions between the microorganism and its host influence the fecal elimination pattern: a low level (<10^3^ CFU/g of feces) and short duration (<10 days) elimination occurs when colonization is limited to the rumen; low levels of elimination are also observed when colonization is extended to the cecum and colon but for longer periods (>30 days) [17].

### 1.3. Zoonotic Spillover

Cattle farming is undoubtedly the major source of environmental contamination from STEC [16], but the pathogens have also been recovered from pigs, goats, deer, horses, dogs, and birds [5,8,13,18]. Previous studies have demonstrated that STEC infections in humans should not be associated only with cattle spillover since there are several proofs of other sources of contamination, like the outbreak of STEC that occurred in Norfolk (UK), which was related to wild rabbits. The high genetic similarity between STEC strains isolated from domestic pets (dogs and cats) and cattle, and the presence of STEC in wildlife animals like red deer and psittacine birds, highlight the possibility that new reservoirs can enhance human exposition and risk of infection [19,20,21,22].

Nonetheless, outbreaks of STEC are generally ascribed to the consumption of contaminated foods of bovine origin, particularly undercooked ground beef patties and unpasteurized milk. For example, in studies of retail ground beef in North America, the prevalence of STEC ranged from 9% to 36.4%, with *E. coli* O157 isolated from 0% to 3.7% of the samples tested [23]. Raw milk and raw milk products are among the main food sources of STEC infection in humans; therefore, identification of pathogens at the herd level is of primary importance for public health [11]. Fecal contamination can be considered the only relevant route to explain the presence of STEC in raw milk. Therefore, the key point in the control of these pathogens is the reduction of fecal contamination of milk [24].

The control of the circulation of STEC on the farm is complex and involves herd management as a whole. Currently, only managerial practices aimed at limiting the presence of STEC in milk are proposed. These measures include: the limitation of the circulation of STEC within the individual farm by hygiene measures (e.g., bedding hygiene, water supplies, alleys cleaning) and the minimization of fecal contamination of milk during milking [25,26].

### 1.4. STEC Detection Methods

In order to obtain laboratory confirmation of STEC infection, one of the following requirements needs to be fulfilled, according to the European Centre for Disease Prevention and Control (ECDC): direct detection of the nucleic acid of *stx1* or *stx2* gene(s) without strain isolation; isolation of nonsorbitol fermenting (NSF) *E. coli* O157 (without testing for Stx or *stx* genes); and isolation/cultivation of an *E. coli* strain that produces Stx or harbors the relative gene(s) [27].

As demonstrated by Dastmalchi et al. [28] and Renter et al. [29], molecular approaches for STEC identification in feces have only been used after bacterial colony isolation on specific plates (e.g., MacConkey agar) from the aforementioned matrix or after an enrichment step of the matrix; no reports of direct molecular analysis on feces samples have been reported. Indeed, after bacterial isolation, two sets of endpoint PCR or real-time PCR are needed to confirm serotype identification and to evaluate the presence of virulence factors that identify *E. coli* serotypes as *E. coli* STEC; the entire procedure is time consuming (55/60 h), even though STEC identification is very precise [30].

Serological methods are commonly used for STEC infection diagnosis; however, even in this case, most of the analysis cannot be performed directly on the sample but requires a prior step of bacterial isolation on agar plates or at least an enrichment step [1]. To date there are several examples of different immunological assays (e.g., traditional ELISA, lateral flow immunoassay, monoclonal antibodies) with common limitations like cross-reaction with other pathogens (i.e., *Brucella abortus*, *Yersinia enterocolitica*, *Vibrio cholera*, *Escherichia hermanni*, *Citrobacter freundii*, *Citrobacter sedlakii*, and *Salmonella*) or even viruses like the two cases of norovirus outbreaks in the United States that yielded false positives for STEC infections [27,31,32,33].

DNA-based methods for researching STEC have the advantage of being rapid and do not require special reagents, such as specific Shiga anti-toxin antisera, or essential equipment for the use of cell cultures. There are numerous PCR methods for the search of *stx1* and *stx2* genes that are capable of detecting all known Shiga toxin subtypes. These tests can be performed both on single bacterial colonies and on mixed cultures, such as enrichment media, or samples such as those shown in [34].

### 1.5. Gap Analysis

Regarding milk and milk products, the World Health Organization in 2018 summarized the main critical points to be considered when establishing surveillance and control programs related to STEC infections and food contaminations. The report, and consequently most of the European national surveillance programs, focus, apart from raw meat and fresh vegetables, on raw milk and raw milk cheeses ready for retail, neglecting the assessment of the sources of contamination at the start of the dairy chain [35] and, more importantly, not identifying the risk factors for the spread of these pathogens within the herd.

This approach is the result of a lack of knowledge (gaps) in preventing the pathogen from spreading in dairy and beef herds at the beginning of the food chain, although the problems associated with STEC/EHEC foodborne disease have been recognized for several years. A recent and useful gap analysis conducted through the Discontools project [36] highlighted the main critical points and gaps that need to be filled regarding STEC surveillance and control. The analysis can be examined on the Discontools website. Among the several issues reported, in our opinion, one of the most important is related to the epidemiologic analysis. In fact, two major gaps in the understanding of STEC epidemiology have been identified: the mechanisms of spreading the infection among herds and how animals are exposed within a farm. Strictly related to this latter issue are gaps in the diagnostic approach. Indeed, new diagnostic approaches and methods are needed to identify mainly non-O157 serotypes in carrier animals and to assess the spread of these serotypes among animals, the contamination of food for human consumption, and human risk related to these foods [36].

### 1.6. Aims of the Pilot Study

The presence of STEC in animals, the severity of the disease in humans, and the role of the environment in maintaining these pathogens support the importance of this group of bacteria in a One Health framework. Moreover, the increasing number of reports on the presence of contaminated foods with STEC serotypes [36], the probable underestimation of these pathogens in dairy herds [11], and the gaps identified related to the epidemiology and detection of these pathogens in dairy herds supported the development of a project to fill these gaps and to develop new approaches to increase the effectiveness of current surveillance programs applied to dairy herds. Within this framework, a pilot study was designed:

1. to assess the feasibility of new molecular methodologies applied to raw milk filters (RMF) as a way to estimate the presence of these pathogens in the herds and to evaluate the application of the same methodologies to calves’ feces, hypothesizing that these animals could play a role in the spread and maintenance of these pathogens in the herd.
2. to apply the same methods to identify the presence of the “Big 7” serotypes in the different types of matrices.

The presence of STEC in calves has been reported in a few studies [37,38,39], but, to the best of our knowledge, this approach was never thought to be a way to identify potential vector animals within and between herds and a potential critical point for control measures. The availability of new commercial molecular assays allows identification of non-O157 serotypes in milk and milk products, simplifying and making the detection process more efficient. However, these methods were not assessed and validated for other biological matrices such as RMF and feces. These validations are pivotal for applying them to a surveillance program based on these matrices.

## 2. Materials and Methods

### 2.1. Herds and Animals

Bulk tank milk (BTM) and RMF samples were collected from 15 different dairy herds in the Lombardy region, whereas fecal samples were collected from calves belonging to three different dairy herds in the Milano province, also in Lombardy. Samples were divided by the location and the time of sampling.

### 2.2. Samples Collection

Milk and filter samples were collected by technicians of the Regional Breeding Association (ARAL) in different areas of Lombardy during routine sampling for milk quality assessment. In-line filters or RMF, made by non-woven fabric, are components of milking machines aimed to catch debris as well as feces particles. The filters are usually changed before milking. For raw milk analysis, about 25 mL of BTM were sampled at the end of the milking, and RMFs were also taken at the end of milking. For each sampling time, both BTM and RMF were sampled in each herd.

Calf feces were collected by herd veterinarians during routine protocols for enteritis prevention. Ten to fifteen g of feces were sampled directly from the rectal ampoule of the animal. Samples were collected in sterile tubes (milk and feces) (VWR international srl, Milano, Italy) and in disposable sterile bags for milk filters. All the samples were immediately frozen (−20 °C), delivered to the laboratories of the Department of Biomedical, Surgical, and Dental Sciences, University of Milan, and kept frozen (−20 °C) until processing.

### 2.3. Samples Preparation

Before the enrichment step, every sample was thawed at room temperature (23 ± 5 °C) inside a laminar flow hood to avoid sample contamination. All samples (raw milk, milk filters, and bovine feces) were prepared for the DNA extraction process following strict sterility procedures to protect the operator from the pathogen and to avoid contaminations that could lead to incorrect results. After thawing, each sample was placed in a Falcon tube (50 mL falcon tube for milk filters and 15 mL falcon tubes for milk and bovine feces) (VWR international srl, Milano, Italy) and enriched with buffered peptone water (BPW) (Biomérieux, Marcy-l’Étoile, France) at a 1:10 ratio, as suggested by the food sample enrichment protocol of real-time PCR producer. The samples were incubated at 37 °C and 5% CO_2_ for 24 h and then 1 mL of the enriched sample was transferred into a 1.5 mL tube to proceed with the extraction step or to be stored in a −20 °C freezer.

### 2.4. DNA Extraction

The DNA extraction process has been carried out with the commercial SureTect^TM^ STEC extraction kit (ThermoFisher Scientific, Waltham, MA, USA). Briefly, 10 µL of proteinase K (ThermoFisher Scientific, Waltham, MA, USA) was added to the side of the SureTect Lysis Tube, then 10 µL of diluted sample was added to the bottom of the tube. The tubes were capped and incubated in a thermoblock at 37 °C for 10 min and then at 95 °C for 5 min. After incubation, the supernatant, containing the sample’s DNA, was used to proceed with the Real-Time PCR assay.

### 2.5. Real-Time PCR Assay

#### 2.5.1. *Escherichia coli* O157:H7 and STEC Virulence Factors Identification

The ThermoFisher Scientific SureTect^TM^ *Escherichia coli* O157:H7 and STEC Screening PCR Assay (ThermoFisher Scientific, Waltham, MA, USA) is based on TaqMan^TM^ PCR technology. Dye-labeled probes target unique DNA sequences specific to STEC. This assay detects STEC *stx*, *eae* genes, and *E. coli* O157:H7 serotype from food and environmental samples. The molecular designs of the primers and probes of this assay are proprietary and cannot be shown. To perform the assay, 20 µL of the sample processed with the SureTect^TM^ STEC extraction kit (ThermoFisher Scientific, Waltham, MA, USA) were loaded in the PCR tube to resuspend the lyophilized master mix already present in the tube. The tubes were capped with optical cap strips and loaded on the Applied Biosystems^TM^ QuantStudio^TM^ 5 Food Safety System (ThermoFisher Scientific, Waltham, MA, USA) to start the real-time PCR run. The results were analyzed using ThermoFisher Scientific RapidFinder^TM^ Analysis Software v1.1. (ThermoFisher Scientific, Waltham, MA, USA). The PCR running conditions consisted of an initial denaturation at 95 °C for 7 min followed by 50 cycles of denaturation at 95 °C for 5 s, annealing and extension at 60 °C for 45 s. The samples that were positive for at least the *stx* gene were processed further with the ThermoFisher Scientific SureTect^TM^ *E. coli* STEC Identification kit (ThermoFisher Scientific, Waltham, MA, USA).

#### 2.5.2. STEC Serotype Identification

The ThermoFisher Scientific SureTect^TM^ *E. coli* STEC (ThermoFisher Scientific, Waltham, MA, USA) Identification commercial kit is used for the rapid qualitative detection of STEC serotypes (O26, O45, O103, O111, O121, O145) from food and environmental safety samples. To perform the assay, 20 µL of the sample processed with the SureTect^TM^ STEC extraction kit (ThermoFisher Scientific, Waltham, MA, USA) were loaded in the PCR tube to resuspend the lyophilized master mix already present in the tube. The tubes were capped with optical cap strips and loaded on the Applied Biosystems^TM^ QuantStudio^TM^ 5 Food Safety System (ThermoFisher Scientific, Waltham, MA, USA) to start the Real Time PCR run. The results were analyzed using ThermoFisher Scientific RapidFinder^TM^ Analysis Software v1.1. (ThermoFisher Scientific, Waltham, MA, USA). The PCR running conditions consisted of an initial denaturation at 95 °C for 7 min followed by 50 cycles of denaturation at 95 °C for 5 s, followed by annealing and extension at 60 °C for 45 s.

### 2.6. Protocol Validation

The diagnostic procedure previously described is aimed at processing food and environmental samples. Therefore, we preliminarily assessed the accuracy of these procedures applied to the different matrices we wanted to investigate (raw milk, raw milk filters, and feces). Following the study of Albonico et al. [11], we artificially contaminated negative samples of raw milk filters and calf feces with specific STEC serotypes (O157, O26, O45, O103, O111, O121, O145), provided by the European Union Reference Laboratory VTEC (ISS Rome, Italy), to assess the assay sensibility of samples different from food matrices. Each sample was inoculated with 10 or 10^2^ CFU of a single *E. coli* serotype, previously grown on Columbia Blood agar (ThermoFisher Scientific, Waltham, MA, USA), suspended in physiologic solution (0.9% NaCl), diluted to the required concentration, and then treated as described above. The negative samples were also tested without artificial contamination to ensure that these kinds of matrices do not yield false positives with this specific assay.

### 2.7. Statistical Analysis

All data were analyzed using SPSS 28.0.1.1 (IBM Corp., Armonk, NY, USA, 2022) and XLSTAT 2023.1.1 (Lumivero, New York, NY, USA). Statistical association between variables has been determined through χ^2^ test and Fisher’s exact test.

## 3. Results

### 3.1. Protocol Validation

The negative controls tested negative for all the target genes in the assay and all the contaminated controls tested positive for the expected virulence and serotype genes at both 10 and 10^2^ CFU inoculum concentrations, enabling us to proceed with the unknown BTM, RMF, and feces.

### 3.2. Data Description

**Figure 1:**
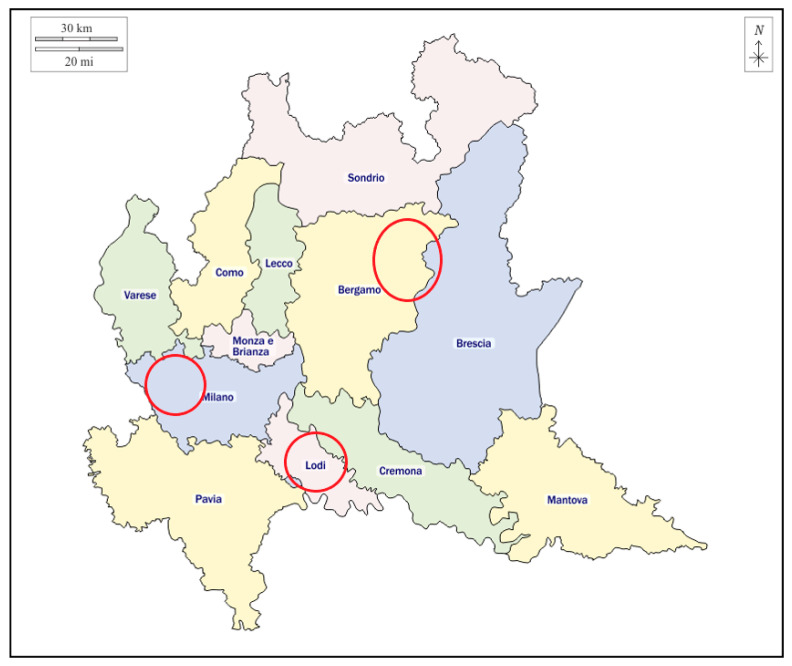
Geographical map of the Lombardy region with areas of sampling (red circles).

A total of 290 samples from 18 different dairy herds were collected and analyzed from January to December 2022 (Figure 1). Of these, 88 were BTM, 104 were RMF, and 98 were calves’ feces samples. Samples have been considered as positive following the principle of maximum precaution and the criterion of direct detection of the nucleic acid of *stx1* or *stx2* gene(s) without strain isolation [27]. All raw results of real-time PCR are provided in Supplementary Table S1.

### 3.3. STEC Virulence Factor Identification

Regarding virulence gene identification, we found three BTM samples positive for the *stx* gene, 10 for the *eae* gene, and no sample positive for both genes. When we considered RMF, a total of six samples tested positive for *stx* presence, 25 for *eae* presence, and 37 for the presence of both genes (Table 1).

When fecal samples were considered, 72 samples were positive for the *stx* gene, 84 for the *eae* gene, and 71 for both genes. For pre-weaning samples, one sample was positive for the *stx* gene, 13 for the *eae* gene, and 71 for both genes; for post-weaning samples, no samples were positive for *stx* or *eae* gene and 35 were positive for both genes. These results are summarized in Table 2.

**Figure 2:**
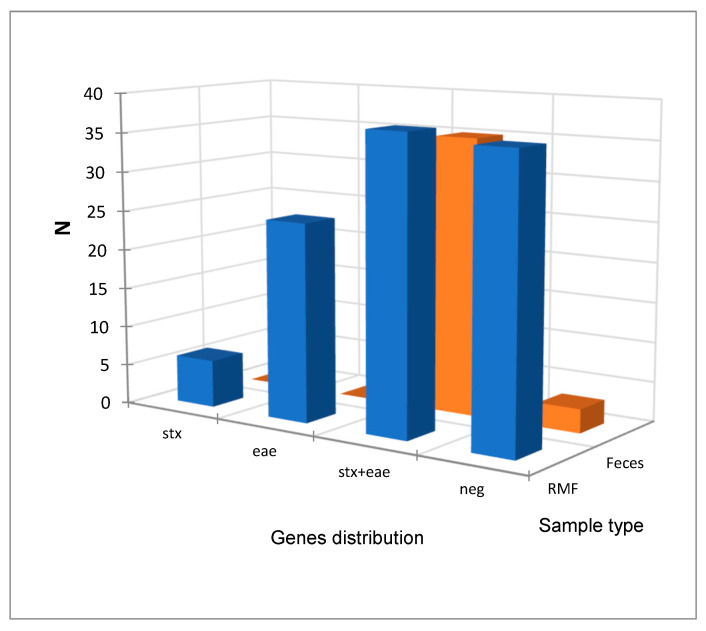
Comparison between the distribution of the virulence genes in raw milk filter (RMF) and feces samples. Blue bars: raw milk filters virulence factors count, orange bars: fecal samples virulence factors count.

The comparison between the distributions of virulence genes in RMF and fecal samples is reported in Figure 2, and the statistical analysis reported in Table 3 showed a statistical difference (α = 0.05) between them, mainly due to a frequency higher than expected in *stx* + *eae* positive fecal samples and, conversely, a lower than expected frequency in RMF samples.

**Figure 3:**
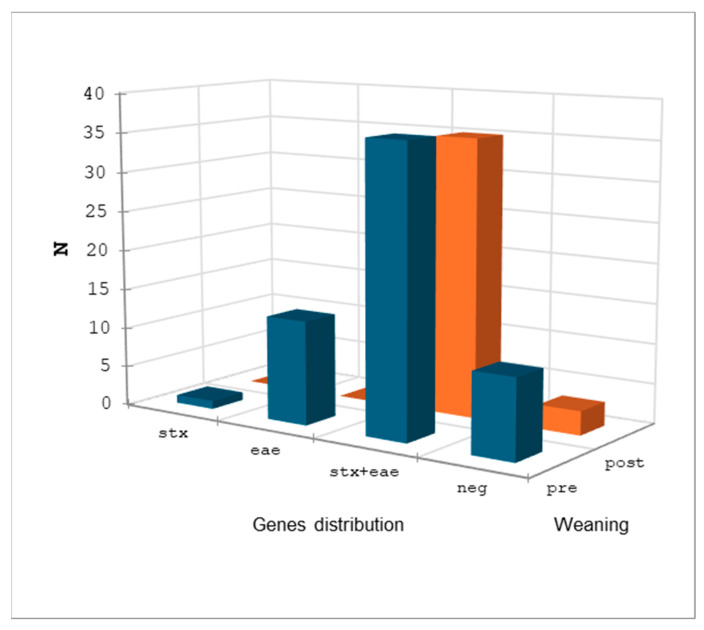
Comparison between the distribution of virulence genes in pre- and post-weaning calf fecal samples. Blue bars: pre-weaning samples virulence factors count, orange bars: post-weaning samples virulence factors count.

Figure 3 reports the comparison between the distributions of virulence genes in fecal samples taken before and after weaning, and Table 4 reports the results of the statistical analysis. The comparison between the distributions of virulence genes in pre- and post-weaning fecal samples (Table 4) showed a statistical difference (α = 0.05) between them, mainly due to a frequency higher than expected in *stx* + *eae* positives in post-weaning fecal samples as well as a lower than expected frequency of *eae* positive samples. As expected, the pattern was reversed in pre-weaning samples.

### 3.4. STEC Serotype Identification

**Figure 4:**
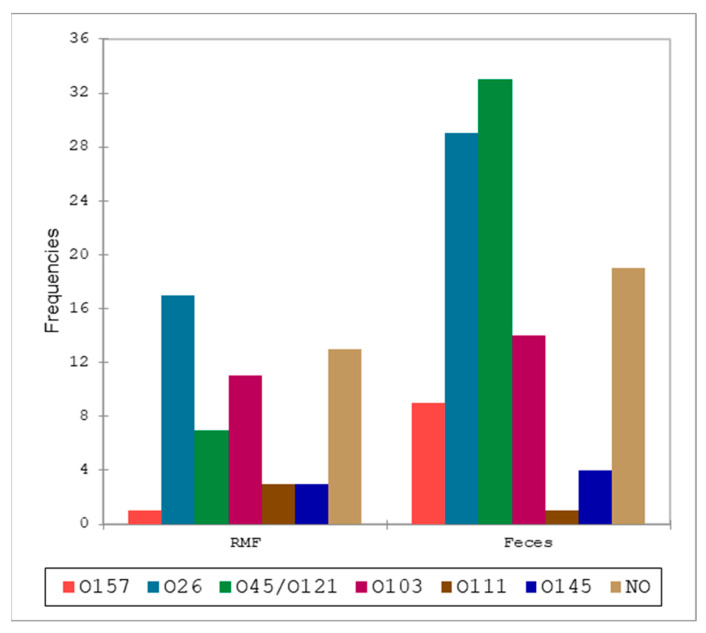
Comparison between the distribution of the serotypes in raw milk filters (RMF) and fecal samples.

A total of 83 (70.3%) of 118 samples positive for the *stx* gene were also positive for at least one STEC serotype. None of the serotypes included in the “Big 7” panel were found in BTM samples, whereas in RMF O157 (*n* = 1), O26 (*n* = 17), O45/O121 (*n* = 7), O103 (*n* = 11), O111 (*n* = 3), and O145 (*n* = 3) were identified. Thirteen *stx*-positive samples from RMF were negative for serotype identification. These results are summarized in Figure 4 and Table 5. The serotype identification of fecal samples led to the identification of O157 (*n* = 9), O26 (*n* = 29), O45/O121 (*n* = 32), O103 (*n* = 14), O111 (*n* = 1), and O145 (*n* = 4) serotypes, while for 19 *stx* positive samples, the serotype was not identified (Table 5).

**Figure 5:**
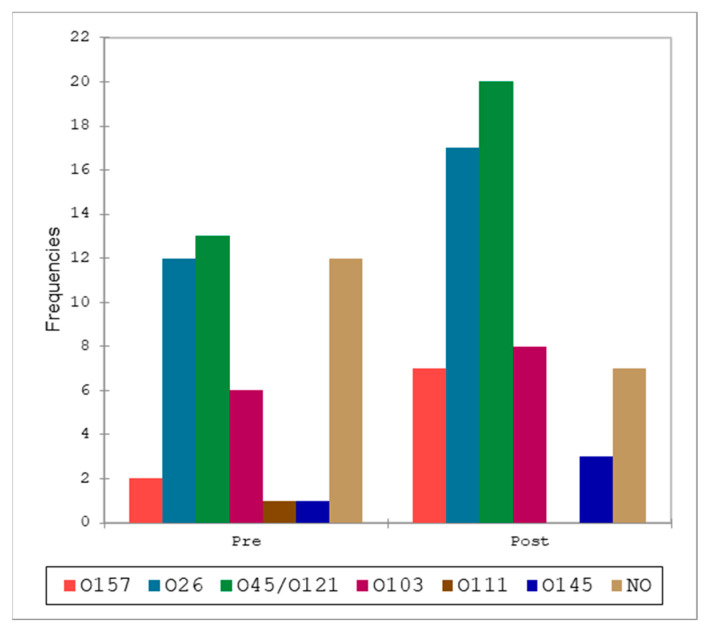
Comparison of the distributions of the serotypes in pre- and post-weaning fecal samples.

All the serotypes identified and classified by type of matrix are reported in Table 5 to visualize relative abundance. The comparison of the serotype distributions (Figure 5 and Table 6) between RMF and feces showed a single significant result with a lower-than-expected frequency of O45/O121 in RMF samples, and the opposite in feces. When pre- and post-weaning distributions were analyzed, we did not find any statistically significant difference at Fisher’s exact test (α = 0.05).

The single percentages do not add up to 100 because some samples were positive for more than one STEC serotype.

## 4. Discussion

STEC represent a serious threat to public health and require an efficient surveillance program to prevent outbreaks in humans. Currently, the only extant surveillance program in Italy, as well as in other countries (e.g., France), involves analysis performed on food (i.e., raw milk, dairy products, raw beef, vegetables) without considering the epidemiologic situation at the herd level, and this program has many flaws regarding methodological and regulatory problems like the primers for molecular identification of the *stx* gene or the definition of a positive sample.

The evidence of several gaps in the current knowledge on the epidemiology of the pathogen, particularly concerning the spread of the infection within the herd, and, therefore, in the surveillance approach at the herd level, support studies aiming to increase our knowledge on this problem. It is important to highlight that future surveillance programs should focus more on virulence factors rather than serotypes since the risk for human illness is mostly related to the *stx* gene. Serotype information is still of value, especially if backed up by a full genomic analysis, since it could lead to a better understanding of the strains’ phylogeny and route of transmission.

The pilot study described in this paper aimed to apply the current available diagnostic kit in matrices different from milk and milk products and to verify if their application may be helpful in the diagnosis of STEC at the herd level (calf feces and RMF). The results confirmed that the commercial kit may be applied to RMF and feces as well as to the target matrices (milk and milk products).

### 4.1. STEC Frequency in Different Matrices

The results of the analysis of BTM, RMF, and calf feces showed a different epidemiological pattern related to the matrix. Indeed, the proportion of positive samples in BTM was very low (3.4% of *stx*-positive samples), whereas the proportion on the RMF of the same herds was higher (41.3% of *stx*-positive samples), as well as in calf feces. Regarding the comparison between RMF and BTM, milk filters were already shown to be a more useful matrix compared to raw milk to identify herd pathogens. Indeed, despite the dimension of the pores in RMF (100–150 μm), they are too big to prevent bacteria from being completely retained by the filter. Previous studies showed their usefulness in identifying pathogens [40,41,42]

Therefore, these differences were expected [11,28,37,42,43] and may be explained as follows:

- Milk: 25 mL of raw milk are sampled from the bulk tank, which is capable of holding 150–10,000 liters of milk at 4 °C, which results in a poor detection level, particularly when the prevalence of STEC-positive cows is very low and/or when milking practices are optimal.
- Milk Filters: with this type of sample, it is easier to find positivity because the main task of the filter is to block and retain any type of fecal or litter debris coming from the milking routine, and all the milk passes through the filter; therefore, there is no dilution effect.

These results support the importance of selecting a proper matrix to monitor the presence of the pathogen at the herd level, like the RMF. Indeed, sampling BTM could lead to an underestimation of the prevalence of this pathogen, and for this reason we suggest that this matrix is not the most appropriate to monitor the presence of STEC at the herd level. Nevertheless, further studies are needed to confirm the use of RFM within a surveillance program for STEC in milk and milk products.

The presence of positive STEC feces in both pre- and post-weaning calves suggests that the infection can be transmitted from positive cows to calves either during calving or by contaminated colostrum and milk. The observed significantly higher-than-expected frequency of *stx* + *eae* genes compared with the RMF frequency supports this hypothesis. The evidence of these ways of transmission also suggests a potential preventive measure based on the use of stored STEC negative milk and colostrum as applied to prevent paratuberculosis transmission [44]. However, how the pathogens enter the herd remains to be elucidated. The most probable ways are the purchase of infected animals (calf, heifer, or cow) and the presence of the pathogens in the environment, such as in water (pools, wells) or fresh forage.

### 4.2. Distribution of Serotypes

The distribution of serotypes among the different samples showed only one significant difference, represented by O45/O121 having a higher-than-expected frequency of these serotypes in feces when compared with RMF. Another result worth mentioning is the low prevalence of O157 isolates representing, overall, less than 20% of the serotypes. Moreover, the most important information arising from these analyses, in our opinion, is represented by the fact that 33 out of 113 (29.2%) samples tested positive for the *stx* virulence gene but negative for the identification of the “Big 7” serotypes.

The current monitoring programs usually consider only the “big five” or the “big seven” without considering other STEC serotypes that may represent a growing threat to public health [30,45].

Following these results, a plausible idea would be to include other common STEC serotypes in the monitoring program, similar to what has been conducted in the work of Capps et al., in order to screen the samples for the other six most common STEC non-top seven serotypes (O2, O74, O109, O131, O168, and O171) and evaluate the prevalence and the resulting burden on public health of these serotypes [45].

## 5. Conclusions

The results obtained further confirmed the usefulness of RMF analysis for the detection of STEC at the herd level. Thus far, our results support the hypothesis that STEC prevalence at the herd level is highly underestimated, and that the surveillance program needs critical and extensive improvements to be more efficient in detecting and preventing STEC infections. Moreover, the presence of STEC in most calf fecal samples and the correspondence between serotype in RMF and feces support the hypothesis of a role of calves in maintaining the infection within the herd. This study aimed to gather information useful to fill the knowledge gap as a preliminary step before designing a proper epidemiological investigation to confirm the role of calves in the epidemiology of these infections. The results of this pilot study also suggest that prevention at the calves’ level may be considered to reduce the risk of spreading the infection within the herd and will support further research projects investigating this aspect of the STEC transmission chain. The epidemiology of these infections and the characteristics of the pathogens clearly show how a One Health approach will be pivotal in improving our capabilities to control the spread of these infections. Finally, more data regarding serotypes and *stx* subtypes should be gathered and made available by the scientific community to better understand the transmission routes of this pathogen and to estimate the risk for severe human illnesses.

## Figures and Tables

**Table 1:** Frequency of *stx* and *eae* genes in bulk tank milk (BTM) and raw milk filter (RMF) samples.

Virulence Gene	BTM Samples N (%)	RMF Samples N (%)
*stx*	3 (3.4%)	6 (5.8%)
*eae*	10 (11.4%)	25 (24%)
*eae* + *stx*	0 (0%)	37 (35.6%)
Negative	75 (85.2%)	36 (34.6%)

**Table 2:** Frequency of *stx* and *eae* genes in calves’ fecal samples.

Virulence Gene	Calves’ Feces N (%)	Pre-Weaning N (%)	Post-Weaning N (%)
*stx*	1 (1%)	1 (1.7%)	0 (0%)
*eae*	13 (13.3%)	13 (21.6%)	0 (0%)
*eae* + *stx*	71 (72.4%)	36 (60%)	35 (92.1%)
Negative	13 (13.3%)	10 (16.7%)	3 (7.9%)

**Table 3:** Results of statistical analysis (Fisher’s exact test) comparing virulence gene distribution between raw milk filters and calf feces samples.

Genes	Raw Milk Filters		Feces	
	Fisher’s Exact Test (P)	Observed vs. Expected Frequency	Fisher’s Exact Test (P)	Observed vs. Expected Frequency
*stx*	0.192	> ^1^	0.192	<
*eae*	0.000	>	0.000	<
*Stx* + *eae*	<0.0001	< ^2^	<0.0001	>
Negative	0.001	>	0.001	<

^1^ higher than expected frequency observed; ^2^ lower than expected frequency observed.

**Table 4:** Results of statistical analysis (Fisher’s exact test) comparing virulence gene distribution between pre- and post-weaning fecal samples.

Genes	Pre-Weaning		Post-Weaning	
	Fisher’s Exact Test (P)	Observed vs. Expected Frequency	Fisher’s Exact Test (P)	Observed vs. Expected Frequency
*stx*	1.000	> ^1^	1.000	<
*eae*	0.001	>	0.001	<
*stx* + *eae*	0.000	< ^2^	0.000	>
Negative	0.360	>	0.360	<

^1^ higher than expected frequency observed; ^2^ lower than expected frequency observed.

**Table 5:** Distribution of STEC serotypes among bulk tank milk (BTM), raw milk filters (RMF), and calf feces positive samples.

Serotype	BTM Samples (%)	RMF Samples (%)	Feces Pre-Weaning (%)	Feces Post-Weaning (%)
O157	0 (0%)	1 (0.9%)	2 (3.3%)	7 (18.4%)
O26	0 (0%)	17 (16.3%)	12 (20%)	17 (44.7%)
O45/O121	0 (0%)	7 (6.7%)	13 (21.6%)	20 (52.6%)
O103	0 (0%)	11 (10.6%)	6 (10%)	8 (21.1%)
O111	0 (0%)	3 (2.9%)	1 (1.6%)	0 (0%)
O145	0 (0%)	3 (2.9%)	1 (1.6%)	3 (7.9%)
NO ^1^	3 (3.4%)	13 (12.5%)	12 (20%)	7 (18.4%)

^1^ Unknown serotype.

**Table 6:** Results of the comparison of serotype distributions between raw milk filters (RMF) and feces samples (Fisher’s exact test).

Serotype	RMF		Feces	
	Fisher’s Exact Test (P)	Observed vs. ExpectedFrequency	Fisher’s Exact Test (P)	Observed vs. Expected Frequency
O157	0.167	< ^1^	0.167	> ^2^
O26	0.584	>	0.584	<
O45/O121	0.013	<	0.013	>
O103	0.254	>	0.254	<
O111	0.110	>	0.110	<
O145	0.688	>	0.688	<
NO1	0.405	>	0.405	<

^1^ lower than expected frequency observed; ^2^ higher than expected frequency observed.

## Data Availability

No new data were created or analyzed in this study.

## Supplementary Materials

The following supporting information can be downloaded at: https://www.mdpi.com/article/10.3390/pathogens13060511/s1, Table S1: raw binary results of real-time PCR analysis.
