# Supplementary material for: How to Improve Surveillance Program for Shiga Toxin-Producing E. coli (STEC): Gap Analysis and Pilot Study
Source: Pathogens. 2024 Jun 17;13(6):511. doi: 10.3390/pathogens13060511 (PMC11206285; doi:10.3390/pathogens13060511)
Supplement: Supplementary file 1 [file pathogens-13-00511-s001.zip › pathogens-2972060-supplementary.pdf]

Table S1: raw binary results of Real-Time PCR analysis (date provided in the format MM/DD/YYYY)

Sample type	Area of collection	Date of collection	*eae*	*stx*	Serotype identified
Raw milk filter	Bergamo province	2/23/2022	-[1]	+[2]	/[3]
Raw milk filter	Bergamo province	2/23/2022	-	-	/
Raw milk filter	Bergamo province	2/23/2022	+	+	O103
Raw milk filter	Bergamo province	2/23/2022	-	-	/
Raw milk filter	Bergamo province	2/23/2022	-	+	/
Raw milk filter	Bergamo province	3/9/2022	-	-	/
Raw milk filter	Bergamo province	3/9/2022	-	-	/
Raw milk filter	Bergamo province	3/9/2022	-	-	/
Raw milk filter	Bergamo province	3/9/2022	-	-	/
Raw milk filter	Bergamo province	3/9/2022	-	-	/
Raw milk filter	Lodi province	3/16/2022	+	-	/
Raw milk filter	Lodi province	3/16/2022	+	-	/
Raw milk filter	Lodi province	3/16/2022	-	-	/
Raw milk filter	Lodi province	3/16/2022	+	-	/
Raw milk filter	Lodi province	4/1/2022	-	-	/
Raw milk filter	Lodi province	4/1/2022	+	+	/
Raw milk filter	Lodi province	4/1/2022	+	+	/
Raw milk filter	Lodi province	4/1/2022	-	-	/
Raw milk filter	Lodi province	4/14/2022	+	+	O26, O45/O121

Raw milk filter	Lodi province	4/14/2022	+	-	/
Raw milk filter	Lodi province	4/14/2022	+	+	/
Raw milk filter	Lodi province	4/14/2022	+	-	/
Raw milk filter	Bergamo province	3/30/2022	-	-	/
Raw milk filter	Bergamo province	3/30/2022	-	-	/
Raw milk filter	Bergamo province	3/30/2022	+	+	O103, O45/O121
Raw milk filter	Bergamo province	3/30/2022	-	-	/
Raw milk filter	Bergamo province	3/30/2022	+	-	/
Raw milk filter	Bergamo province	4/13/2022	+	-	/
Raw milk filter	Bergamo province	4/13/2022	-	-	/
Raw milk filter	Bergamo province	4/13/2022	+	+	O103
Raw milk filter	Bergamo province	4/13/2022	+	+	O26
Raw milk filter	Bergamo province	4/13/2022	-	-	/
Raw milk filter	Bergamo province	4/13/2022	-	-	/
Raw milk filter	Bergamo province	4/27/2022	+	-	/
Raw milk filter	Bergamo province	4/27/2022	-	-	/
Raw milk filter	Bergamo province	4/27/2022	-	-	/
Raw milk filter	Bergamo province	4/27/2022	+	+	O26

Raw milk filter	Bergamo province	4/27/2022	-	-	/
Raw milk filter	Lodi province	5/9/2022	+	-	/
Raw milk filter	Lodi province	5/9/2022	+	+	O103, O145
Raw milk filter	Lodi province	5/9/2022	+	+	O145
Raw milk filter	Lodi province	5/9/2022	+	-	/
Raw milk filter	Lodi province	5/17/2022	+	-	/
Raw milk filter	Lodi province	5/17/2022	+	-	/
Raw milk filter	Lodi province	5/17/2022	+	-	/
Raw milk filter	Lodi province	5/17/2022	-	-	/
Raw milk filter	Bergamo province	5/11/2022	-	-	/
Raw milk filter	Bergamo province	5/11/2022	-	-	/
Raw milk filter	Bergamo province	5/11/2022	-	-	/
Raw milk filter	Bergamo province	5/11/2022	+	+	/
Raw milk filter	Bergamo province	5/11/2022	-	-	/
Raw milk filter	Lodi province	5/25/2022	+	-	/
Raw milk filter	Lodi province	5/25/2022	+	+	O26, O103, O145
Raw milk filter	Lodi province	5/25/2022	+	+	O26
Raw milk filter	Lodi province	5/25/2022	+	+	O26
Raw milk filter	Bergamo province	5/11/2022	-	-	/
Raw milk filter	Lodi province	5/31/2022	+	+	O26
Raw milk filter	Lodi province	5/31/2022	+	-	/
Raw milk filter	Lodi province	5/31/2022	+	+	/
Raw milk filter	Lodi province	5/31/2022	+	+	O26, O45/O121

Raw milk filter	Lodi province	6/28/2022	+	-	/
Raw milk filter	Lodi province	6/28/2022	+	-	/
Raw milk filter	Lodi province	6/28/2022	+	+	O26
Raw milk filter	Lodi province	6/28/2022	+	+	O26
Raw milk filter	Bergamo province	6/7/2022	+	+	O26
Raw milk filter	Bergamo province	6/7/2022	+	+	/
Raw milk filter	Bergamo province	6/7/2022	-	-	/
Raw milk filter	Bergamo province	6/7/2022	+	+	O103, O45/O121
Raw milk filter	Bergamo province	6/22/2022	-	-	/
Raw milk filter	Bergamo province	6/22/2022	+	-	/
Raw milk filter	Bergamo province	6/22/2022	-	-	/
Raw milk filter	Bergamo province	6/22/2022	-	+	O103
Raw milk filter	Bergamo province	7/6/2022	-	+	O26
Raw milk filter	Bergamo province	7/6/2022	-	-	/
Raw milk filter	Bergamo province	7/6/2022	+	-	/
Raw milk filter	Bergamo province	7/6/2022	-	+	O103
Raw milk filter	Lodi province	7/26/2022	+	+	O26, O45/O121
Raw milk filter	Lodi province	7/26/2022	+	-	/
Raw milk filter	Lodi province	7/26/2022	+	+	O26
Raw milk filter	Lodi province	7/26/2022	+	+	O26

Raw milk filter	Lodi province	9/21/2022	+	+	/
Raw milk filter	Lodi province	9/21/2022	+	+	O111
Raw milk filter	Lodi province	9/21/2022	-	-	/
Raw milk filter	Lodi province	9/21/2022	+	+	/
Raw milk filter	Bergamo province	7/27/2022	-	-	/
Raw milk filter	Bergamo province	7/27/2022	+	-	/
Raw milk filter	Bergamo province	8/10/2022	+	+	O103
Raw milk filter	Bergamo province	8/10/2022	+	+	/
Raw milk filter	Bergamo province	8/24/2022	-	-	/
Raw milk filter	Bergamo province	8/24/2022	-	-	/
Raw milk filter	Bergamo province	8/24/2022	-	-	/
Raw milk filter	Lodi province	10/26/2022	+	+	O26, O111, O45/O121
Raw milk filter	Lodi province	10/26/2022	+	+	O26, O103, O111
Raw milk filter	Lodi province	10/26/2022	-	-	/
Raw milk filter	Lodi province	10/26/2022	+	-	/
Raw milk filter	Milan province	4/4/2022	-	+	/
Raw milk filter	Milan province	4/4/2022	+	+	O157
Raw milk filter	Milan province	5/5/2022	+	-	/
Raw milk filter	Milan province	5/27/2022	-	-	/
Raw milk filter	Milan province	5/27/2022	+	+	O45/O121
Raw milk filter	Milan province	5/27/2022	+	+	O103
Raw milk filter	Milan province	6/24/2022	+	-	/
Raw milk filter	Milan province	6/24/2022	+	-	/

Raw milk filter	Milan province	7/22/2022	+	+	/
Raw milk	Bergamo province	2/23/2022	-	-	/
Raw milk	Bergamo province	2/23/2022	+	-	/
Raw milk	Bergamo province	2/23/2022	-	-	/
Raw milk	Bergamo province	2/23/2022	-	-	/
Raw milk	Bergamo province	2/23/2022	-	-	/
Raw milk	Bergamo province	3/9/2022	+	-	/
Raw milk	Bergamo province	3/9/2022	-	-	/
Raw milk	Bergamo province	3/9/2022	-	-	/
Raw milk	Bergamo province	3/9/2022	-	-	/
Raw milk	Bergamo province	3/9/2022	-	-	/
Raw milk	Lodi province	3/16/2022	-	-	/
Raw milk	Lodi province	4/1/2022	-	-	/
Raw milk	Lodi province	4/14/2022	-	-	/
Raw milk	Bergamo province	3/30/2022	-	-	/
Raw milk	Bergamo province	3/30/2022	+	-	/
Raw milk	Bergamo province	3/30/2022	-	-	/
Raw milk	Bergamo province	3/30/2022	-	-	/

Raw milk	Bergamo province	3/30/2022	-	-	/
Raw milk	Bergamo province	3/30/2022	-	-	/
Raw milk	Bergamo province	4/13/2022	-	-	/
Raw milk	Bergamo province	4/13/2022	-	-	/
Raw milk	Bergamo province	4/13/2022	-	-	/
Raw milk	Bergamo province	4/13/2022	-	-	/
Raw milk	Bergamo province	4/13/2022	-	-	/
Raw milk	Bergamo province	4/13/2022	-	-	/
Raw milk	Bergamo province	4/13/2022	-	-	/
Raw milk	Lodi province	5/9/2022	-	-	/
Raw milk	Lodi province	5/17/2022	-	-	/
Raw milk	Bergamo province	4/27/2022	-	-	/
Raw milk	Bergamo province	4/27/2022	+	-	/
Raw milk	Bergamo province	4/27/2022	-	-	/
Raw milk	Bergamo province	4/27/2022	-	-	/
Raw milk	Bergamo province	4/27/2022	-	-	/
Raw milk	Bergamo province	4/27/2022	-	-	/
Raw milk	Bergamo province	5/11/2022	-	-	/

Raw milk	Bergamo province	5/11/2022	-	-	/
Raw milk	Bergamo province	5/11/2022	-	-	/
Raw milk	Bergamo province	5/11/2022	-	-	/
Raw milk	Bergamo province	5/11/2022	-	-	/
Raw milk	Bergamo province	5/11/2022	-	+	/
Raw milk	Bergamo province	5/11/2022	-	-	/
Raw milk	Lodi province	5/31/2022	-	-	/
Raw milk	Lodi province	6/28/2022	-	-	/
Raw milk	Bergamo province	6/7/2022	-	-	/
Raw milk	Bergamo province	6/7/2022	-	-	/
Raw milk	Bergamo province	6/7/2022	-	+	/
Raw milk	Bergamo province	6/7/2022	-	-	/
Raw milk	Bergamo province	6/7/2022	-	-	/
Raw milk	Bergamo province	6/7/2022	-	-	/
Raw milk	Bergamo province	6/22/2022	-	-	/
Raw milk	Bergamo province	6/22/2022	-	-	/
Raw milk	Bergamo province	6/22/2022	+	-	/
Raw milk	Bergamo province	6/22/2022	-	-	/

Raw milk	Bergamo province	6/22/2022	-	-	/
Raw milk	Bergamo province	6/22/2022	-	-	/
Raw milk	Bergamo province	7/6/2022	-	-	/
Raw milk	Bergamo province	7/6/2022	-	-	/
Raw milk	Bergamo province	7/6/2022	-	-	/
Raw milk	Bergamo province	7/6/2022	-	-	/
Raw milk	Bergamo province	7/6/2022	-	-	/
Raw milk	Bergamo province	7/6/2022	-	-	/
Raw milk	Lodi province	7/26/2022	-	-	/
Raw milk	Lodi province	9/21/2022	-	-	/
Raw milk	Bergamo province	7/27/2022	-	-	/
Raw milk	Bergamo province	7/27/2022	-	-	/
Raw milk	Bergamo province	7/27/2022	-	-	/
Raw milk	Bergamo province	8/10/2022	-	-	/
Raw milk	Bergamo province	8/10/2022	-	-	/
Raw milk	Bergamo province	8/10/2022	-	-	/
Raw milk	Bergamo province	8/24/2022	-	-	/
Raw milk	Bergamo province	8/24/2022	-	-	/

Raw milk	Bergamo province	8/24/2022	-	-	/
Raw milk	Lodi province	10/26/2022	-	-	/
Raw milk	Milan province	4/4/2022	-	-	/
Raw milk	Milan province	4/4/2022	-	-	/
Raw milk	Milan province	4/4/2022	-	+	/
Raw milk	Milan province	5/5/2022	-	-	/
Raw milk	Milan province	5/5/2022	+	-	/
Raw milk	Milan province	5/5/2022	+	-	/
Raw milk	Milan province	5/27/2022	-	-	/
Raw milk	Milan province	5/27/2022	-	-	/
Raw milk	Milan province	5/27/2022	+	-	/
Raw milk	Milan province	6/24/2022	-	-	/
Raw milk	Milan province	6/24/2022	+	-	/
Raw milk	Milan province	6/24/2022	+	-	/
Raw milk	Milan province	7/22/2022	-	-	/
Raw milk	Milan province	7/22/2022	-	-	/
Raw milk	Milan province	7/22/2022	-	-	/
Bovine feces Post-weaning	Milan province	4/4/2022	+	+	/
Bovine feces Post-weaning	Milan province	4/4/2022	-	-	/
Bovine feces Post-weaning	Milan province	4/4/2022	-	-	/
Bovine feces Pre-weaning	Milan province	4/4/2022	+	+	/
Bovine feces Pre-weaning	Milan province	4/4/2022	+	+	O45/0121
Bovine feces Pre-weaning	Milan province	4/4/2022	+	+	O45/O121, O103

Bovine feces Pre-weaning	Milan province	4/4/2022	+	+	/
Bovine feces Pre-weaning	Milan province	4/4/2022	+	+	O103
Bovine feces Pre-weaning	Milan province	4/4/2022	+	+	O103
Bovine feces Pre-weaning	Milan province	4/4/2022	+	+	O26, O103
Bovine feces Pre-weaning	Milan province	4/4/2022	+	+	O26
Bovine feces Post-weaning	Milan province	4/4/2022	+	+	/
Bovine feces Post-weaning	Milan province	4/4/2022	+	+	/
Bovine feces Post-weaning	Milan province	4/4/2022	+	+	/
Bovine feces Post-weaning	Milan province	4/4/2022	+	+	/
Bovine feces Pre-weaning	Milan province	4/4/2022	-	-	/
Bovine feces Pre-weaning	Milan province	4/4/2022	+	-	/
Bovine feces Pre-weaning	Milan province	4/4/2022	+	+	/
Bovine feces Pre-weaning	Milan province	4/4/2022	+	+	/
Bovine feces Pre-weaning	Milan province	4/4/2022	+	+	/
Bovine feces Post-weaning	Milan province	4/4/2022	+	+	O103, O26, O45/O121
Bovine feces Post-weaning	Milan province	4/4/2022	+	+	O26, O45/O121
Bovine feces Post-weaning	Milan province	4/4/2022	+	+	O157, O26, O45/O121

Bovine feces Pre-weaning	Milan province	5/5/2022	+	+	/
Bovine feces Pre-weaning	Milan province	5/5/2022	+	+	/
Bovine feces Pre-weaning	Milan province	5/5/2022	+	-	/
Bovine feces Pre-weaning	Milan province	5/5/2022	+	+	O45/0121
Bovine feces Post-weaning	Milan province	5/5/2022	+	+	O45/0121
Bovine feces Post-weaning	Milan province	5/5/2022	+	+	/
Bovine feces Pre-weaning	Milan province	5/5/2022	-	-	/
Bovine feces Pre-weaning	Milan province	5/5/2022	+	+	O26, O103, O45/O121
Bovine feces Pre-weaning	Milan province	5/5/2022	+	+	O26
Bovine feces Pre-weaning	Milan province	5/5/2022	+	+	O26
Bovine feces Post-weaning	Milan province	5/5/2022	+	+	/
Bovine feces Post-weaning	Milan province	5/5/2022	+	+	O26, O103, O45/O121
Bovine feces Post-weaning	Milan province	5/5/2022	+	+	O26, O103
Bovine feces Pre-weaning	Milan province	5/5/2022	-	-	/
Bovine feces Pre-weaning	Milan province	5/5/2022	+	-	/
Bovine feces Pre-weaning	Milan province	5/5/2022	+	+	O26, O45/O121
Bovine feces Post-weaning	Milan province	5/5/2022	+	+	O26

Bovine feces Post-weaning	Milan province	5/5/2022	+	+	O157, O26, O45/O121
Bovine feces Post-weaning	Milan province	5/5/2022	+	+	O26
Bovine feces Pre-weaning	Milan province	5/27/2022	+	+	/
Bovine feces Pre-weaning	Milan province	5/27/2022	+	-	/
Bovine feces Pre-weaning	Milan province	5/27/2022	+	+	/
Bovine feces Pre-weaning	Milan province	5/27/2022	+	+	O26
Bovine feces Pre-weaning	Milan province	5/27/2022	+	+	/
Bovine feces Pre-weaning	Milan province	5/27/2022	+	+	O26
Bovine feces Pre-weaning	Milan province	5/27/2022	+	+	O26
Bovine feces Pre-weaning	Milan province	5/27/2022	+	+	O145, O45/O121
Bovine feces Post-weaning	Milan province	5/27/2022	+	+	O145, O45/O121
Bovine feces Post-weaning	Milan province	5/27/2022	+	+	O145
Bovine feces Post-weaning	Milan province	5/27/2022	+	+	O26, O103, O145, O45/O121
Bovine feces Pre-weaning	Milan province	5/27/2022	+	-	/
Bovine feces Pre-weaning	Milan province	5/27/2022	+	-	/
Bovine feces Pre-weaning	Milan province	5/27/2022	+	-	/
Bovine feces Pre-weaning	Milan province	5/27/2022	-	-	/

Bovine feces Post-weaning	Milan province	5/27/2022	+	+	O103, O45/O121
Bovine feces Post-weaning	Milan province	5/27/2022	+	+	O45/O121
Bovine feces Post-weaning	Milan province	5/27/2022	+	+	O103, O45/O121
Bovine feces Pre-weaning	Milan province	6/24/2022	+	+	O45/O121
Bovine feces Pre-weaning	Milan province	6/24/2022	+	+	O103, O45/O121
Bovine feces Pre-weaning	Milan province	6/24/2022	+	-	/
Bovine feces Pre-weaning	Milan province	6/24/2022	+	+	O45/O121
Bovine feces Pre-weaning	Milan province	6/24/2022	+	+	O45/O121
Bovine feces Post-weaning	Milan province	6/24/2022	+	+	O157, O45/O121
Bovine feces Post-weaning	Milan province	6/24/2022	+	+	O157, O26, O45/O121
Bovine feces Post-weaning	Milan province	6/24/2022	+	+	O157, O45/O121
Bovine feces Pre-weaning	Milan province	6/24/2022	-	-	/
Bovine feces Pre-weaning	Milan province	6/24/2022	-	+	/
Bovine feces Pre-weaning	Milan province	6/24/2022	+	-	/
Bovine feces Pre-weaning	Milan province	6/24/2022	+	+	O26
Bovine feces Post-weaning	Milan province	6/24/2022	+	+	O26
Bovine feces Post-weaning	Milan province	6/24/2022	+	+	O26, O45/O121

Bovine feces Pre-weaning	Milan province	6/24/2022	-	-	/
Bovine feces Pre-weaning	Milan province	6/24/2022	+	+	O157
Bovine feces Pre-weaning	Milan province	6/24/2022	-	-	/
Bovine feces Post-weaning	Milan province	6/24/2022	+	+	O157
Bovine feces Post-weaning	Milan province	6/24/2022	+	+	O157, O103, O45/O121
Bovine feces Pre-weaning	Milan province	7/22/2022	-	-	/
Bovine feces Pre-weaning	Milan province	7/22/2022	+	+	O45/O121
Bovine feces Pre-weaning	Milan province	7/22/2022	+	+	/
Bovine feces Pre-weaning	Milan province	7/22/2022	+	+	O157, O26, O45/O121
Bovine feces Post-weaning	Milan province	7/22/2022	+	+	O26, O45/O121
Bovine feces Post-weaning	Milan province	7/22/2022	-	-	/
Bovine feces Post-weaning	Milan province	7/22/2022	+	+	O26, O45/O121
Bovine feces Pre-weaning	Milan province	7/22/2022	+	-	/
Bovine feces Pre-weaning	Milan province	7/22/2022	+	-	/
Bovine feces Pre-weaning	Milan province	7/22/2022	-	-	/
Bovine feces Pre-weaning	Milan province	7/22/2022	+	-	/
Bovine feces Post-weaning	Milan province	7/22/2022	+	+	O26

Bovine feces Post-weaning	Milan province	7/22/2022	+	+	O26, O45/O121
Bovine feces Pre-weaning	Milan province	7/22/2022	+	+	O26, O111
Bovine feces Pre-weaning	Milan province	7/22/2022	-	-	/
Bovine feces Pre-weaning	Milan province	7/22/2022	+	+	O45/O121
Bovine feces Pre-weaning	Milan province	7/22/2022	+	-	/
Bovine feces Post-weaning	Milan province	7/22/2022	+	+	O26
Bovine feces Post-weaning	Milan province	7/22/2022	+	+	O103, O45/O121

[1] Negative result for the Real-Time PCR detection of the target gene

[2] Positive result for the Real-Time PCR detection of the target gene

[3] No serotype identification
